# Practical study on the application of full-cycle fast track surgical nursing model in patients with replantation of severed fingers: A retrospective analysis

**DOI:** 10.1097/MD.0000000000035665

**Published:** 2023-10-20

**Authors:** Hui Ju, Ling Li, Xiangyun Wang, Jing Mu, Lei Ge, Shumin Wu

**Affiliations:** a Department of Emergency, People’s Hospital of Rizhao, Jining Medical University, Shandong, China; b School of Nursing, Sun Yat Sen University, Guangdong, Guangzhou, China; c Nursing Department, People’s Hospital of Rizhao, Jining Medical University, Shandong, China.

**Keywords:** full-cycle fast track surgical nursing model, functional recovery, quality of life, replantation of severed fingers

## Abstract

To explore the effect of full-cycle fast track surgical (FTS) nursing in patients with replantation of severed fingers, and observe its effect on functional recovery of replanted fingers and quality of life of patients. From January 2021 to December 2022, 86 patients with replantation of severed fingers were selected from Rizhao People’s Hospital, 41 patients were given routine perioperative care, 45 patients were given full-cycle rapid rehabilitation surgical care. Compare the relevant indexes of the 2 groups of patients during hospitalization. Three months after discharge, the finger function recovery of the 2 groups were compared, and the quality of life of the patients was scored with the QL-Index scale, and the satisfaction was evaluated at the same time. The first time of getting out of bed and the time of hospitalization in the full-cycle FTS nursing group were significantly shorter than those in the conventional nursing group, and the incidence of postoperative nausea, vomiting, constipation and venous thromboembolism were significantly lower than those in the conventional nursing group. The anxiety score was significantly lower than that in the conventional nursing group, the difference was statistically significant (*P* < .05). There was no significant difference in the incidence of arteriovenous crisis between the 2 groups (*P* > .05). Three months after discharge, the scores of finger sensation and movement, quality of life and satisfaction of patients in the FTS nursing group were higher than those in the conventional nursing group, and the difference was statistically significant (*P* < .05). Full-cycle fast track surgical nursing model can improve the in-patient experience, reduce the incidence of complications, promote rapid rehabilitation, improve the quality of life of patients, and improve satisfaction.

## 1. Introduction

Due to the change in the mode of social production and the nature of work, the incidence of hand injuries has increased year by year. As one of the important organs of the human body, the hand can complete all kinds of daily fine movements, which is the key to ensure normal work, and life. When the finger is severed due to trauma, replantation of the severed finger can effectively restore the shape and function of the affected finger, which is the most important treatment strategy at this stage. However, the survival rate and functional recovery of severed fingers are not only dependent on the successful implementation of replantation surgery, but also the rehabilitation nursing intervention.^[1]^ At present, full-cycle fast track surgical (FTS) nursing has been tried to be used in the field of orthopedic trauma.^[2]^ This model aims to reduce the stress response of patients during the perioperative period and accelerate the rehabilitation process of patients. With the continuous improvement of patients requirements for functional recovery of replanted fingers, since January 2022, the hand surgery department of Rizhao People’s Hospital has taken FTS and continuous nursing as the guiding theory, and has applied evidence-based medicine throughout the whole process of patients perioperative, inpatient and post-discharge rehabilitation, providing scientific, accurate, and high-quality nursing measures to maximize the recovery of the functions of the fingers. It will improve and develop the disability prevention strategy and full-cycle functional protection in the clinical and scientific research practice of finger rehabilitation. Based on this, this study uses a retrospective analysis method to analyze the clinical data of 86 patients with replantation of severed fingers who were treated in the hand surgery of Rizhao People’s Hospital from January 2021 to December 2022, and to explore the application effect of the full-cycle fast track surgical nursing model. The specific report is as follows.

## 2. Materials and methods

### 2.1. Clinical data

A retrospective analysis was made of 102 patients who underwent replantation of severed fingers in hand surgery of Rizhao People’s Hospital from January 2021 to December 2022. In 2021, the routine nursing mode was applied, with a total of 49 patients, 41 survived, and the survival rate of replantation of severed fingers was 83.67%. In 2022, the full-cycle FTS nursing mode was applied. A total of 53 patients, 45 survived, and the survival rate of replantation of severed fingers was 84.91%. This experiment has been reviewed and approved by the Medical Ethics Committee of Rizhao People’s Hospital. The ethical approval for this series of cases was obtained by the Ethics Committee of the People’s Hospital of Rizhao City, Shandong Province, China (Approval No. 2020110201). All patients signed informed consent. All clinical investigations were conducted according to the principles expressed in the declaration of Helsinki.

#### 2.1.1. Inclusion criteria.

1. Conforming to the diagnosis and treatment criteria of severed finger injury.
2. Patients who underwent replantation of severed fingers within 10 hours after injury.
3. Those with normal cognitive function and the ability to act independently.
4. Patients with severed fingers survived.

#### 2.1.2. Exclusion criteria.

1. Patients with massive limb injury or mental disorder.
2. Patients who did not come to the hospital on time for further diagnosis and lost contact.
3. Treat patients who are transferred or discharged halfway.

According to the inclusion and exclusion criteria, this study will be divided into routine nursing group (41 cases) and full-cycle FTS nursing group (45 cases) according to whether the full-cycle FTS nursing mode is applied. There was no statistically significant difference in general data such as gender, age, education level and cause of injury between the 2 groups (*P* > .05). The comparison of the general data was shown in Table 1.

**Table 1:** The comparison of the general data.

Data classification	Routine nursing group (n = 41)	Full-cycle FTS nursing group (n = 45)	x^2^/t	*P* value
Gender				
Male	34	36	0.12	.73
Female	7	9		
Age	47.61 ± 11.14	45.18 ± 12.07	0.97	.33
Educational level				
Primary and below	13	17	0.64	.73
Junior high school to senior high school	16	14		
College and above	12	14		
Injury causes				
Electric saw injury	7	16	4.91	.18
Crushing injury	11	6		
Incised injury	13	12		
Avulsion injury	10	11		

There was no statistically significant difference in general data such as gender, age, education level and cause of injury between the two groups (*P* > .05).

FTS = full-cycle fast track surgical.

### 2.2. Research methods

Routine nursing group: monitor the patient’s vital signs during the perioperative period, inform the patient of the precautions after the operation, routine anesthesia nursing after the operation, and guide the patient to recover and exercise. The patient was reexamined in the outpatient department 1, 2, and 3 months after discharge, and the outpatient doctor gave the patient rehabilitation training guidance.

Full-cycle FTS nursing group: on the basis of routine nursing measures, an intervention group is composed of competent doctors, anesthesiologists, surgical nurses, ward nurses, rehabilitation nurses, psychological consultants, and nutritionists to develop detailed full-cycle FTS nursing measures for patients.

Full-cycle FTS care measures: Preoperative nursing: the responsible nurse in the ward introduced the patient’s condition, operation process, and possible complications after the operation to the patient and his family members in detail, evaluated the patient’s psychological state and gave psychological counseling to eliminate the patient’s fear of the operation; Two hours before the operation, the patient was advised to drink a proper amount of water; Antibiotics were used 30 minutes before operation; Intraoperative nursing: The anesthesiologist accurately administered anesthetic drugs and selected low-dose high-effective anesthetic drugs; The operating nurse should control the temperature of the operating room at 25°C, strengthen the warm care, closely monitor the changes of the patient’s vital signs, and maintain smooth breathing; Instruct the patient to maintain the correct surgical position, gently massage the affected limb every 30 minutes to ensure smooth blood circulation; Control fluid input and minimize fluid input while maintaining vital signs; Postoperative nursing: maintain the temperature of the ward at 26°C, keep the affected finger under continuous light irradiation treatment, and ensure the temperature of the affected finger unchanged when opening the window for ventilation every day; After the operation, Wada drinking water test was carried out. According to the results, the patients were given the corresponding diet type. The nutritionist matched the patients with high-protein and digestible diet, encouraged the patients to eat more fresh fruits, vegetables and coarse fiber diet to ensure the smooth defecation, and urged the patients to drink water not < 1500 mL per day to promote blood circulation; Strengthen nursing patrol, guide patients to correct supine posture and the method of turning over and changing position, to avoid the pressure of the affected limb affecting the venous return; Special education on postoperative precautions for patients and their families, including preventive measures for postoperative complications, and anti-smoking education; Psychological nursing: psychological nursing runs through the whole process, timely assessing the psychological state of patients, encouraging patients to express their feelings, teaching patients to relax pain training, timely taking pain measures, reducing patients anxiety and depression, and improving bad mood; Rehabilitation nursing: patients were encouraged to get out of bed and move 5 days after operation, and the affected limbs were suspended and fixed by forearm sling; 8 days after the operation, the patients were instructed to exercise the wrist and upper limb joints, 3 times a day, 5 to10 minutes each time; Two weeks after the operation, the affected wrist was actively extended and flexed, the affected finger was passively moved, and the intensity of movement was increased from small to large. The interphalangeal joint underwent isometric contraction and isometric contraction exercise, twice a day, 5 minutes to 10 minutes each time; Three weeks after operation, guide the patient to actively bend and extend the wrist joint, 5 to 6 times a day, 30 minutes each time; After 6 to 12 weeks, the patients were given sensory function training. The method was to gently slide a thin rod (such as a toothpick) along the palmar side of the finger from the proximal end of the section to the distal end to ask the patients how they felt until the patients could distinguish the stimulation of different shapes and textures; After the fracture is well healed, pull out the Kirschner wire and guide the patient to perform active and passive flexion and extension of the affected finger. For those with limited flexion and extension activities, elastic bandage in flexion position and elastic splint in extension position can be used for fixation, 3 to 4 times a day, 30 to 60 minutes each time; In addition, patients were guided to exercise the grasping and kneading abilities of replanted fingers step by step through actions such as dividing beans, kneading plasticine, and pulling elastic band.

### 2.3. Evaluation indicators

#### 2.3.1. Postoperative recovery.

Time of first eating and first getting out of bed: the time of first eating is determined by the standard of no nausea and vomiting after eating. The first time of getting out of bed activity is determined by the standard that the blood circulation of the affected finger does not change after the first time of getting out of bed activity; Complications: Complications include vascular crisis, venous thromboembolism, nausea, vomiting, and constipation. Among them, vascular crisis includes arterial crisis and venous crisis; Venous thromboembolism includes deep venous thrombosis and pulmonary embolism; Constipation refers to patients who have not defecated or have difficulty in defecating more than 3 days after surgery after normal diet; GAD-7 Generalized Anxiety Disorder Scale was routinely used to assess anxiety in patients 7 days after operation; The hospitalization days of the 2 groups were compared.

#### 2.3.2. Finger function recovery.

Three months after discharge, the degree of finger function recovery was evaluated, including sensory function evaluation and motor function evaluation.

1. Finger sensory function evaluation: Finger sensory recovery is divided into 5 levels according to the evaluation standard of the British Medical Research Association.^[3]^ Grade S4: finger sensation returned to normal, 2-point resolution < 6 mm; Grade S3: recovery of finger touch and superficial sensation, and existence of 2-point discrimination; S2 grade: slight recovery of superficial sensation of fingers; S1 grade: finger skin pain recovery; Grade S0: no sensation in fingers.
2. Evaluation of hand motor function: The motor function of the affected finger was evaluated according to the Trial Standard for the Evaluation of Partial Function of Upper Limbs of the Society of Hand Surgery of the Chinese Medical Association.^[4]^ Excellent: The patient’s finger joint activity is normal without any adverse phenomenon; Good: finger function is < 75% of that of the healthy side, and more than 50% of that of the healthy side; Poor: finger function is < 50% of that of the healthy side.

#### 2.3.3. Patient quality of life score.

Three months after discharge, QL-Index scale was used to evaluate the quality of life of patients, including daily life, activity ability, health, support, and life feeling. Each dimension was scored 0 to 2 points, with a total score of 10 points. The higher the score, the better the quality of life.

#### 2.3.4. Patient satisfaction score.

Likert 5-grade scoring method was used to score the satisfaction degree. The nursing satisfaction degree was divided into satisfactory, relatively satisfactory, general, less satisfactory, and dissatisfied, corresponding to 5, 4, 3, 2, and 1 point respectively.

### 2.4. Statistical analysis method

The statistical software SPSS26.0 was used to process the relevant data. The measurement data of this study meet the normal distribution and variance homogeneity test, which are described by means and standard deviation, and compared by student's *t*-test. The counting data were described by the number of cases and compared by chi-square test. Student's *t*-test was used to compare the time of first eating, the time of first getting out of bed and the time of discharge, anxiety score and patients' quality of life between the two groups. The nausea and vomiting, constipation, arteriovenous crisis, venous thromboembolism, sensory and motor function of the two groups were compared by chi-square test. The inspection level is α = 0.05, *P* < .05 is statistically significant.

## 3. Results

### 3.1. Comparison of relevant indexes after operation

By comparing and analyzing the relevant indexes of the 2 groups of patients after operation, it was found that the time of first eating, the time of first getting out of bed and the time of discharge of the patients in the full-cycle FTS nursing group were significantly less than those in the conventional nursing group, the incidence of nausea, vomiting, constipation and venous thromboembolism was lower than that in the conventional nursing group, and the anxiety score was significantly lower than that in the conventional nursing group. The above differences were statistically significant (*P* < .05); There was no significant difference in the incidence of arteriovenous crisis between the 2 groups (*P* > .05). The comparison of postoperative related indicators is shown in Table 2.

**Table 2:** The comparison of postoperative related indicators.

Data classification	Routine nursing group (n = 41)	Full-cycle FTS nursing group (n = 45)	x^2^/z	*P* value
Time of first eating (x ± s, h)	6.32 ± 0.47	2.13 ± 0.81	−8.21	<.01
Time of first getting out of bed (x ± s, d)	7.49 ± 0.68	5.36 ± 0.48	−8.12	<.01
Time of discharge (x ± s, d)	14.46 ± 3.54	12.42 ± 3.22	−2.54	.01
Nausea/vomiting	13	5	5.50	.02
Constipation	15	4	9.56	<.01
Arteriovenous crisis	7	11	0.70	.40
Venous thromboembolism	11	4	4.80	.03
Anxiety score	10.80 ± 3.15	9.24 ± 3.45	−2.60	<.01

The first time of getting out of bed and the time of hospitalization in the full-cycle FTS nursing group were significantly shorter than those in the conventional nursing group, and the incidence of postoperative nausea, vomiting, constipation and venous thromboembolism were significantly lower than those in the conventional nursing group. The anxiety score was significantly lower than that in the conventional nursing group, the difference was statistically significant (*P* < .05). There was no significant difference in the incidence of arteriovenous crisis between the two groups (*P* > .05).

d = days, FTS = full-cycle fast track surgical, h = hours.

### 3.2. Comparison of recovery of hand function

When the patients were investigated for recovery of hand function 3 months after discharge, the motor function and sensory function recovery of patients in the full-cycle FTS care group were significantly better than those in the usual care group, and the differences were statistically significant (*P* < .05). The comparison of postoperative recovery of hand function is shown in Table 3.

**Table 3:** The comparison of postoperative recovery of hand function.

Data classification	Motor function		Sensory function		
	Excellent	Good	Poor	<3	≥3
Routine nursing group (n = 41)	4	20	17	24	17
Full-cycle FTS nursing group (n = 45)	12	29	4	15	30
x^2^	13.54		5.50		
*P* value	<.01		.02		

The motor function and sensory function recovery of patients in the full-cycle FTS care group were significantly better than those in the usual care group, and the differences were statistically significant (*P* < .05).

FTS = full-cycle fast track surgical.

### 3.3. Comparison of patients quality of life

Patients were evaluated 3 months after discharge using the QL-index scale, and the quality of life of patients in the full-cycle FTS care group was significantly better than that of the usual care group, with statistically significant differences (*P* < .05). The comparison of quality of life is shown in Table 4.

**Table 4:** The comparison of quality of life.

Data classification	Routine nursing group (n = 41)	Full-cycle FTS nursing group (n = 45)	x^2^/z	*P* value
QL-index	7.71 ± 1.01	8.42 ± 0.89	−3.42	<.01

The quality of life of patients in the full-cycle FTS care group was significantly better than that of the usual care group, with statistically significant differences (*P* < .05).

FTS = full-cycle fast track surgical.

### 3.4. Comparison of patient satisfaction

A survey of patient satisfaction 3 months after discharge found that patient satisfaction in the full-cycle FTS care group was significantly better than that in the usual care group, and the difference was statistically significant (*P* < .05). The comparison of patient satisfaction is shown in Table 5.

**Table 5:** The comparison of quality of life.

Data classification	Routine nursing group (n = 41)	Full-cycle FTS nursing group (n = 45)	x^2^/z	*P* value
Patient satisfaction	2.78 ± 0.79	4.13 ± 0.76	−6.16	<.01

Patient satisfaction in the full-cycle FTS care group was significantly better than that in the usual care group, and the difference was statistically significant (*P* < .05).

FTS = full-cycle fast track surgical.

### 3.5. Patient recovery process

**Figure 1.**
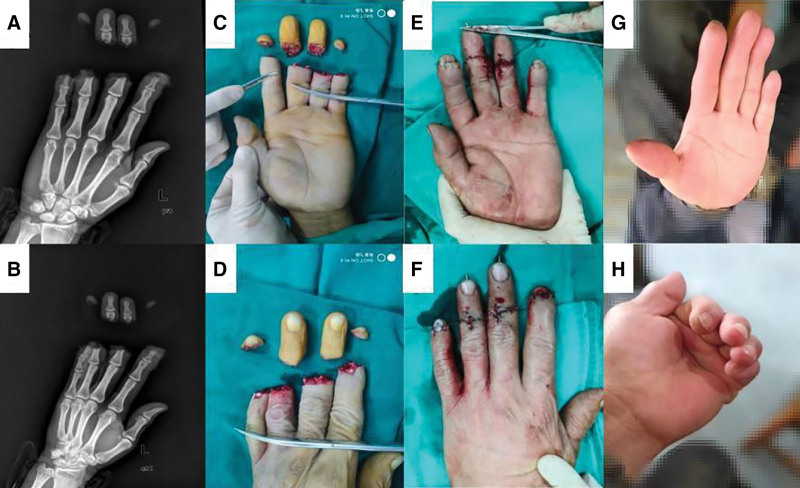
Patient recovery process. (A and B) Complete interruption of the bony interposition of fingers. (C and D) Complete disconnection of fingers. (E and F) Revascularization was good after reimplantation surgery. (G and H) Reviewed in third month after operation, recovering well.

The images of the patient with severed finger reconstruction were shown in Figure 1. the patient’s left suggestive finger, middle finger, ring finger, and little finger were completely dissociated, the patient underwent immediate reimplantation surgery, the procedure was uneventful, and the patient’s hand function recovered well 3 months after surgery.

## 4. Discussion

With the development of microsurgery technology, replantation has become the best treatment strategy to restore the shape and function of the hand of patients with severed fingers. How to shorten the patient’s hospitalization time, reduce the incidence of postoperative complications and restore the hand function on the basis of ensuring the survival of replanted fingers is the focus of the current hand surgery rehabilitation nursing field. In the past, patients were given routine postoperative care, focusing on the survival of replanted fingers, while ignoring the management of patients own feelings and rehabilitation functions.^[5]^ Based on the theory of rapid rehabilitation surgical nursing, this study implemented FTS nursing during the perioperative period to ensure the success of surgery and reduce the physical and psychological stress response of patients. After discharge, continuous nursing intervention was given to the patients, so as to timely understand the recovery of the patients and give patient guidance, which met the nursing needs of the patients in the recovery stage. This full-cycle nursing intervention has achieved good results in clinical practice.

Based on the clinical data, the survival rate of replantation of severed fingers in the conventional nursing group and the full-cycle FTS nursing group was 83.67% and 84.91%, and the survival rate of replantation of severed fingers in the full-cycle FTS nursing group was slightly higher than that in the conventional nursing group, but the difference was not statistically significant, indicating that the full-cycle FTS nursing mode had no adverse effect on the survival of the affected fingers, and whether it could effectively improve the survival rate of the affected fingers still needed to be further verified by increasing the sample size of the study. After comparing the relevant indexes of the patients with severed finger replantation, it was found that the time of first eating, time of getting out of bed and time of discharge of the patients in the full-cycle FTS nursing group were earlier than those in the conventional nursing group. The incidence of postoperative nausea, vomiting, constipation and venous thromboembolism was significantly lower than that in the conventional nursing group. The reason may be that the full-cycle FTS nursing shortened the fasting time and absolute bedtime of the patients by optimizing the anesthesia process and post-operation process. Early postoperative ambulation can accelerate blood circulation, increase cardiac stroke volume, and facilitate thoracic movement, improve vital capacity and prevent postoperative lung infection; Early postoperative ambulation can accelerate the muscle contraction of the lower limbs, thus promoting the venous blood flow of the lower limbs, preventing the formation of venous thrombosis of the lower limbs, and is conducive to the postoperative recovery; Early ambulation after operation can improve the blood circulation of gastrointestinal tract, accelerate the peristalsis of gastrointestinal tract, and facilitate the recovery of gastrointestinal function.^[6]^In addition, pain will cause the body to release a variety of injury factors, cause vasospasm and induce vascular crisis.^[7]^ Full-cycle FTS nursing group advocates timely pain assessment and analgesia after surgery, which can not only ensure the comfort of patients, but also promote wound healing, and to some extent alleviate the anxiety of patients, which is more conducive to early recovery. In addition, compared with the conventional nursing group, the probability of occurrence of arteriovenous crisis in the full-cycle FTS nursing group did not increase significantly. It was laterally verified that early postoperative ambulation was not the direct cause of arteriovenous crisis in the fingers, and also suggested that clinical medical staff should be familiar with all the risk factors leading to the occurrence of vascular crisis, and give corresponding intervention measures to reduce the occurrence of arteriovenous crisis.^[8]^The recovery of hand function of patients in the full-cycle FTS nursing group was significantly better than that in the conventional nursing group 3 months after operation, which may be related to the use of stepwise functional exercise after operation. Combining the rehabilitation requirements at different stages after operation with the actual situation of patients, and guiding the patients to conduct functional exercise scientifically is conducive to preventing tendon adhesion and joint stiffness after operation, and also conducive to promoting the conversion of basic movements to fine movements of replanted fingers.^[9]^In addition, the full-cycle FTS nursing group tracks the rehabilitation care of patients after discharge, further consolidating the treatment effect. The improvement of the treatment effect will bring qualitative improvement to the quality of life of patients, and at the same time, the satisfaction will also be correspondingly improved.

## 5. Conclusion

The full-cycle fast track surgical nursing model for reimplanted patients based on perioperative FTS care and continuation care after discharge in this study is able to accelerate the speed of patient recovery, reduce complications, better restore hand function, and accelerate the normalization of patients lives and jobs, which is of great significance to promote patients physical and psychological rehabilitation and deserves further promotion in the clinic.

## Author contributions

**Conceptualization:** Ling Li, Xiangyun Wang.

**Data curation:** Ling Li, Xiangyun Wang.

**Formal analysis:** Ling Li, Shumin Wu.

**Funding acquisition:** Shumin Wu.

**Investigation:** Jing Mu, Shumin Wu.

**Methodology:** Xiangyun Wang.

**Project administration:** Hui Ju, Lei Ge.

**Resources:** Lei Ge.

**Software:** Jing Mu, Shumin Wu.

**Supervision:** Hui Ju, Jing Mu, Shumin Wu.

**Validation:** Hui Ju, Jing Mu, Shumin Wu.

**Writing – original draft:** Hui Ju, Ling Li, Lei Ge.

**Writing – review & editing:** Hui Ju.
